# Motivation System of Healthy and Safe Nutrition in Iranian Adolescent Girls: A Qualitative Study

**DOI:** 10.1155/2021/5556759

**Published:** 2021-07-12

**Authors:** Mohammad Hossein Kaveh, Leila Moradi, Mohammad Ali Morowatisharifabad, Azadeh Najarzadeh, Hossein Fallahzadeh

**Affiliations:** ^1^Research Center for Health Sciences, Institute of Health, Department of Health Promotion, School of Health, Shiraz University of Medical Sciences, Shiraz 71557, Iran; ^2^Social Determinants of Health Research Center, Department of Health Promotion, School of Health, Shahid Sadoughi University of Medical Sciences, Yazd 89151, Iran; ^3^Elderly Health Research Center, Department of Health Promotion, School of Health, Shahid Sadoughi University of Medical Sciences, Yazd 89151, Iran; ^4^Nutrition and Food Security Research Center, Department of Nutrition, School of Health, Shahid Sadoughi University of Medical Sciences, Yazd 89151, Iran; ^5^Research Center of Prevention and Epidemiology of Non-Communicable Disease, Department of Biostatistics and Epidemiology, School of Health, Shahid Sadoughi University of Medical Sciences, Yazd 89151, Iran

## Abstract

**Objective:**

Motivation determines the possibility of an individual's intention to accept health-related behaviors. Despite the important role of the motivational system in conducting healthy and safe nutrition behavior, this issue has not been adequately addressed. The purpose of this qualitative study was to explain the healthy and safe nutrition motivation system among Iranian adolescent girls.

**Method:**

In this qualitative study, conventional content analysis was used and participants were selected using the purposive sampling method. Semistructured in-depth interviews were conducted with 42 adolescent girls in Shiraz, Iran. The interviews continued until data saturation was obtained. Qualitative data were analyzed using the Graneheim and Lundman qualitative content analysis by MAX-Q-DA (version 10) software.

**Results:**

The data analysis resulted in three main categories for the participants' motivational factors for healthy and safe nutrition: (1) maintaining health and social functions, (2) maintaining the family's mental and economic health, and (3) achieving goals and success in life. The first category included two subcategories: “desire to have an appropriate look and appearance” and “fear of diseases and their complications.” The second category included two subcategories of “maintaining the family's mental health” and “maintaining the family's economic health.” The third category consisted of two subcategories: “desire to form a family and be successful in life” and “achieving the future career goals.”

**Conclusion:**

The motivation to maintain health and social functions, to maintain the family's mental and economic health, and to achieve goals and be successful in life has a powerful impact on the decisions and behaviors of adolescent girls to have safe and healthy nutrition. Therefore, recognizing the motivational system, as a key and determinative factor in developing the adolescents' intention to adopt health-related behaviors, is an effective step to design and develop successful interventions in promoting nutritional behaviors.

## 1. Introduction

Nowadays, nutrition is a global challenge for adolescents' health, so that rapid changes in lifestyle and dietary patterns have exposed them to various diseases associated with unhealthy and unsafe nutrition, for example, diseases transmitted through contaminated foods and chronic diseases such as obesity, diabetes, cardiovascular disease, stroke, hypertension, osteoporosis, dental diseases, and some types of cancers [1, 2].

The prevalence of inappropriate nutritional behaviors in adolescents continues into adulthood [1]. Therefore, the adults' nutritional behavior can be changed and improved from adolescence. Since adolescent girls will play a key role with regard to nutrition and food safety in the family, improving their nutritional behavior can lead to the health and well-being of themselves and their family [3].

Various factors can affect the nutritional behaviors of adolescents. Identification of the individual intent development probability determinants to adopt the health-related behaviors, including healthy and safe nutrition behaviors, plays an important role in designing and developing the educational interventions for promoting nutritional behaviors. Therefore, determination of the effective factors on adolescents' nutrition behaviors can increase the success of interventions designed to improve the nutritional behaviors [4].

Another determinant of the individual intent development probability to adopt health-related behaviors is the motivational system. The motivation refers to the behaviors associated with the purpose and its psychological roots. It also creates some forces of pressure and attraction that lead people towards the desired achievements and lets them avoid them the unintended consequences. The motivational system is a combination of different drives, emotional states, values, and attitudes with different emotional weight. These emotional factors create a certain level of arousal that leads the individuals to a specific measure and intention [5]. Various studies examined the determinants of the nutritional behavior based on the cognitive-social patterns of health behavior and behavioral change, such as planned behavior theory, social-cognitive theory, and health belief model [6–8].

### 1.1. Object

Although motivation can act as a major driving force in adolescents to adopt healthy and safe nutrition behaviors, no study has ever investigated the motivational system related to the healthy and safe nutrition behavior. Furthermore, sociocultural and environmental factors in society can cause a significant difference between adolescents' experiences, which can be examined using a qualitative approach. Therefore, this study was conducted using a qualitative approach to explain the healthy and safe nutrition motivation system among adolescent girls in Iran.

## 2. Materials and Methods

### 2.1. Study Design

This study was conducted in Shiraz City, Iran, and lasted from October 2018 to March 2019. In this qualitative study, a conventional content analysis approach was adopted to explain the healthy and safe nutrition motivational system among Iranian adolescent girls.

### 2.2. Participants

Participants included 42 girls in the age range of 13-15 years selected from the schools in four education districts of Shiraz, Iran. The purposeful sampling method was applied with maximum variation in terms of the demographic characteristics of the participants' family, including age, educational level, occupational status, number of children, and family income (Table 1).

To conduct the research, female students of the eighth grade, who attended public schools in Shiraz City, were selected. Participants should have the required ability and willingness to communicate with the interviewer. They also were required to sign the informed consent forms to participate in the study and have enough information about the topic in question.

Considering the academic profile and the teachers' judgment as primary criteria, a number of students were selected as potential informants. Then, the students who showed, in the interview process, relatively good communication skills, were active in sharing information, and knew nutritional facts from their current curriculum were considered as acceptable informants. In contrast, students who, despite the interviewer's efforts and facilitation (e.g., using more probing questions), were poor at sharing ideas and unable to recall nutritional facts (not having enough information) were excluded from the study (*N* = 3).

### 2.3. Data Collection

The data were collected using semistructured, in-depth, individual interviews based on some predefined guidance questions. The interviews were conducted in the students' schools in a quiet place to ensure the participants' privacy. Each interview lasted about 40 to 75 minutes.

Before the interviews, the researcher introduced herself and outlined the study goals for the participants, instructions on how the interview was conducted and how the voice recorder was applied for recording the interviews, confidentiality of the information, and possibility of withdrawal from the study at any stage of the research with no need to explain the reason. Finally, the volunteer participants were required to sign the informed consent forms to participate in the research. All the interviews were recorded, and sampling continued until data saturation was reached. Although no new conceptual information was obtained after 35 interviews, seven more interviews were conducted to ensure the data saturation, which resulted in no new information. The interview questions included demographic information and basic research questions based on the motivational system structure, including “What does motivate you to care for your nutrition and have safe and healthy food behaviors?” and “What are the important and valuable factors for you to have healthy and safe food behaviors?” The follow-up questions were also asked by the researcher to gain insight into the participants' experiences, for example, “Can you explain your experiences with an example?” and “Please explain a bit more.”

### 2.4. Data Analysis

The Lundman and Graneheim approach was applied to analyze the data [9]. All interviews were recorded using a digital recorder and transcribed verbatim. In order to identify the semantic units of each category, all manuscripts were carefully and repeatedly reviewed by the researchers. In the next stage, the semantic units were summarized and converted into codes. Later, the initial coding was performed in the text. In the next stage, the sub-subcategories, subcategories, and main categories were derived in accordance with the similarities and differences among the codes. The MAX-Q-DA software (version 10) was run to manage the data analysis. All stages of data analysis were monitored and controlled by the research supervisors and advisers.

### 2.5. Data Trustworthiness and Rigor

In this research, four criteria of Lincoln and Guba, including credibility, transferability, confirmability, and dependability, were applied to ensure the trustworthiness and rigor of the data [10]. The research team devoted enough time to collect data by providing the participants with enough time to express their experiences. They maintained their prolonged engagement with the study by frequent review of the information and immersion in the data. In order to confirm the credibility of the analyzed content, a number of encoded texts were returned to a number of participants for member check. Data dependability was confirmed by external check (by some professionals who were not engaged in the data collection process) and research team members (the supervisor and advisor). To hit this target, these professionals were provided with the encoded manuscripts; they were asked to review, discuss, and reach a consensus over the final codes. Moreover, the researcher increased the transferability of the information by describing the participants and sampling method, as well as the data collection time and location in details. In order to increase the confirmability and dependability of the data, researchers precisely described the study details and recorded the data collection processes (transcription, encoding, and analysis).

### 2.6. Ethics Approval and Consent to Participate

To conduct this study, the ethical approval was received from the Ethics Committee of the Public Health School in Shahid Sadoughi University of Medical Sciences, Yazd, Iran (Reference Number http://ir.ssu.sh.rec.1397.071/), in compliance with the Helsinki Declaration. Before the interviews, the researcher introduced herself and provided the participants and their parents with the research objectives, instructions on how the interview was conducted and how the voice recorder was applied for recording the interviews, confidentiality of the information, and possibility of withdrawal from the study at any stage of the research with no need to explain the reason. Then, parents were asked to sign an informed consent form if they agree that their children participate in this study. Finally, the volunteer participants were required to sign the consent forms.

## 3. Results

Students' values motivated them to consider their nutritional behaviors, which were classified under three categories, six subcategories, and 12 sub-subcategories (Table 2).

### 3.1. Maintaining Health and Social Functions

The participants' desire to maintain their normal health and common functions in the community acted as a driving force for them to consider the foods' health and safety. The motivation to preserve normal health and common functions included two subcategories.

#### 3.1.1. Desire to Have an Appropriate Look and Appearance

The motivation to have an appropriate look and appearance encouraged the adolescent girls to consider their nutritional behaviors. This desire was derived from two subcategories.

*(1) Desire to Have Fitness*. The participants felt uncomfortable about obesity and poor fitness; they wanted to have an appropriate level of body fitness. Some girls who were overweight or had obesity stated that their poor body fitness caused some problems for them in society.

“I feel embarrassed because of my fat body; I cannot do the things that I like. If I participate in social activities, people will make fun of me.” (Participant 23)

*(2) Desire to Have Beautiful Skin and Hair*. Participants wanted to have a good look and appearance among their friends and classmates. In this regard, they mentioned long beautiful hair and smooth skin without any smudges as some criteria of beauty. They reported that when their skin had acne, they felt very bad about themselves.

“Once, because I ate fatty foods, especially chips and seeds, I bad acnes on my face, I was really ugly. My friends laughed at me.” (Participant 3)

#### 3.1.2. Fear of Diseases and their Complications

Participants were afraid of the diseases and their consequences caused by unhealthy and unsafe nutrition. Their fears included two sub-subcategories.

*(1) Fear of Medical Complications following the Diseases*. This fear was caused by food poisoning, chronic diseases (such as diabetes, cardiovascular disease, and hypertension), dietary restrictions, hospitalization, need for frequent tests, and extreme treatments.

“I like to eat all my favorite food, but when I get blood sugar like my cousin, I cannot eat some foods.” (Participant 14)

*(2) Fear of Disrupting Social Functions*. Students noted many implications that could be caused by the diseases associated with inappropriate nutritional behaviors. They believed that these consequences created problems in their academic performance, such as school absenteeism and academic failure. Furthermore, diseases disrupted their daily activities including their sporting and artistic activities in society and separated them from friends.

“I am worried that if I get sick, I cannot participate in the exercises or team works with my friends; so, I will be separated from my friends and lose them.” (Participant 5)

### 3.2. Maintaining the Family's Mental and Economic Health

Participants believed that their decreased health status due to inappropriate nutritional behaviors had bad consequences for the family. In other words, family members were worried and their mental health was threatened. Furthermore, the treatment costs were high and affected the economic health of the family. Therefore, the subcategories of maintaining the mental and economic health of the family were considered as the most important motivational factors for having appropriate nutritional behaviors presented by the participants.

#### 3.2.1. Maintaining Mental Health of the Family

Participants believed that inappropriate nutritional behaviors not only damaged their own physical and mental health but also endangered the mental health of their family. They pointed out that making mood changes and disrupting family functions were among the factors that affected their mental health.

*(1) No Change of Mood in the Family*. All participants emphasized that families liked to have healthy and happy children. They will be distressed and worried when they see that their child's health is in danger seriously. In this case, they even suffer from depression and disappointment.

Participants stated that their interest in the family and their unwillingness to cause changes in the family mood were among the prominent motivating factors to have appropriate nutritional behaviors.

“When I get sick, my family members are worried, especially if they know that I have to take medicines for a long time. They might be disappointed and depressed.” (Participant 31)

*(2) No Disturbance in Family Functions*. The participants believed that in the case of a disease incidence, their care and treatment program would create limitations for their family. For example, the disease may restrict their relationships with friends and relatives. Family members should change their diet and omit some of their favorite foods or sweeteners. In addition, diseases can disrupt the favorite activities of the family, such as recreational travel programs.

As the participants reported, their interest in the family and their unwillingness to interfere with the family functions were another motivating factor for them to have appropriate nutritional behaviors.

“If I hospitalized due to a disease, my family's most time will be spent on me. They will not have enough time to have fun or visit the relatives and friends.” (Participant 27)

#### 3.2.2. Maintaining the Economic Health of the Family

Participants stated that if they encountered a serious problem due to inappropriate nutritional behaviors, treatment and medical care would impose high costs on the family. They mentioned the two subcategories.

*(1) Not Causing Job Problems for the Family*. The participants with employed parents believed that if they developed a disease due to inappropriate nutritional behaviors, they would put their parents in trouble. They explained that their parents must leave their work to take care of their child; as a result, taking many leaves may lead to reduced salary or job loss and make financial problems for the family.

Participants reported that they were reluctant to create such situations for their family; therefore, not causing job problems for the parents or other family members was another motivational factor that led the individuals toward the appropriate nutritional behaviors.

“Once, I got sick with salty stick crackers. It was so hard for my mother to take care of me because she works and the employer does not allow her to leave.” (Participant 18)

*(2) Not Imposing Medical Expenses on the Family*. Participants stated that their illness not only imposed heavy costs of care and treatment (such as medication, tests, and surgery) but also wasted the family savings or budgets dedicated to the recreational and travel programs.

As the adolescents reported, “not imposing medical expenses on the family” was an important motivator for them to pay more attention to their nutritional behaviors.

“If I take blood sugar, I have to receive insulin every day; my family should spend a lot to buy the insulin.” (Participant 25)

### 3.3. Achieving Goals and Success in Life

As the participants noted, appropriate nutritional behaviors played an important role in achieving goals in the future and being successful in their marital life. The motivation to achieving goals and success in life included two subcategories:

#### 3.3.1. The Desire to Form a Family and Be Successful in Life

Participants reported that they wanted to maintain their health and beauty in order to form a family in the future. They mentioned the two sub-subcategories:

*(1) The Desire to Marry and Be Successful in Married Life*. The other motivational factors that lead the participants to perform the appropriate nutritional behaviors were “willingness to have beautiful body and clear skin to find the right spouse” and “to maintain health for perfuming the duties of married life.”

“When I grow up, I like to have fine skin and beautiful body to find a good husband; then, I will not be worried that I do not look good and people do not choose me.” (Participant 29)

*(2) Fear of Infertility and Disorder in Pregnancy*. Participants believed that inappropriate nutritional behaviors could lead to infertility and disorder in their future pregnancies. They referred to impaired pregnancy cases, such as having no baby, giving birth to weak children, stillbirth and dead fetus in the womb, infertility due to ovarian or uterine cysts, and delayed pregnancy due to obesity as their motivational factors to conduct appropriate nutritional behaviors.

“I really want to have a baby when I grow up. If I know what I eat may have negative effects on my pregnancy, I will not consume those foods.” (Participant 28)

“I eat very few sausages and salami, I'm afraid will get ovarian cysts and be unable to have a baby. Additives are added to these products to prevent them from spoiling.” (Participant 4)

“Every time my dad goes to the store, my mom urges him to buy these chickens and eggs of green that are not injected to them drugs and hormones, they are healthier and do not cause infertility.” (Participant 7)

#### 3.3.2. Achieving the Future Career Goals

Participants reported that the “desire to have a desired job” and “fear of unemployment due to disease complications” were two factors that encouraged them to pay more attention to their nutritional behaviors.

*(1) Desire to Have a Desired Job*. According to the participants' view points, if they wanted to reach their desired jobs in the future and be successful in that job, they should keep their health. For example, they said in order to be a pilot, one needs to be tall and should have healthy eyes, which requires appropriate nutritional behaviors. Therefore, the desire to reach the desired job was another motivational factor raised by the participants.

“I know that I should be tall in order to pass the pilots' test. So, I started drinking milk every day to help my bones' growth.” (Participant 20)

*(2) Fear of Unemployment due to Disease Complications*. Participants believed that some diseases created physical problems that prevented them from achieving the desired job. In this regard, they mentioned fears such as height shortness, disability, or loss of body organs due to a disease as the motivational factors to have appropriate nutritional behaviors.

“Sometimes I'm really worried about my obesity. I fear of heart problems that I cannot work, and become a burden on my family in the future.” (Participant 10)

## 4. Discussion

The present study was conducted for the first time in Shiraz City to explain the motivational system of Iranian adolescent girls with regard to safe and healthy eating habits. The aim of this study was to explain the motivational factors in nutritional behaviors from the viewpoint of eighth-grade high school female students.

The findings showed that three key factors of “maintaining health and social functions,” “achieving goals and success in life,” and “maintaining the family's mental and economic health” were very important for students. These three factors were considered as the determinant motivational factors in intent development probability to adopt healthy and safe nutrition behaviors.

Factors of being healthy and having the ability to do social and daily activities created the required force and pressure on the students to perform appropriate nutritional behaviors. Students emphasized that unhealthy and unsafe nutritional behaviors not only endangered their health but also disrupted their social and routine activities. One of the most challenging health concerns was the desire to have an appropriate look and appearance. Students believed that having beautiful body, skin, and hair played a significant role in creating a pleasant look and appetite and would be attractive to friends, peers, and the opposite sex. Earlier studies also reported that the desire of the adolescent girls towards weight loss and fitness was due to their interest in having an appropriate appearance, being attractive, and gaining acceptance among friends [11, 12]. In the present study, some students felt uncomfortable about their large and fat body, which was resulted from overeating or consuming fastfood and fatty foods. They mentioned that obesity not only affected health in adulthood but also created some problems throughout life, such as having inappropriate appearance, being limited in choosing the right clothes, undermining the self-esteem, being under pressure due to peers' mocking, and reducing group activities. In previous studies, adolescents, especially girls, were unhappy with their obesity and felt embarrassed and hate. Their most important reasons were inappropriate appearance, limitation in choosing clothes, and loss of fit body [13, 14].

Another concern with regard to the health maintenance was the fear of diseases and their complications resulting from inappropriate nutritional behaviors. Students believed that the diseases could cause consequences for them, including medical conditions and disruptions in social functions. The need for long-term treatment or surgical procedures, hospitalization, dietary constraints, school absenteeism, and disruption in the academic performance or other daily activities were considered as other disease complications mentioned by the students. In line with these findings, previous research on diabetes and its side effects caused by inappropriate food habits reported various unexpected medical and health problems, such as infection, hospitalization, frequent absence from school, and disruptions in school and out-of-school activities [15]. Maintenance of the mental and economic health of the family was another driver for appropriate nutritional behaviors in the students. They believed that if their health was endangered by poor nutritional behaviors, their families would suffer from economic and psychological harms, which was very annoying and disturbing for the adolescents. One of the participants' desires with regard to the mental health maintenance of the family was not changing the family mood; they mentioned that their diseases caused discomfort, concern, despair, and depression among the family members. Previous studies also mentioned the adolescents' concern about the effect of their disease on the mood, anxiety, depression, and stress of their family members. As a result, the students tried to prevent changes in the mood of the family [16, 17].

With regard to maintenance of the family mental health, the other aim of the students was not disturbing the family functions. They believed that their disease caused by inappropriate nutrition limited the relationship of the family members with others and disturbed the favorite recreational activities of the family such as travel programs. In another study, adolescents referred to the impact of the disease on family planning [18].

Given the maintenance of the economic health of the family, the students tried to avoid causing occupational problems for their parents by observing their nutritional health. They believed that in the case of diseases, the family was involved in the care and treatment process and parents were forced to leave their jobs, which could threaten their job position and cause financial problems. In line with these findings, a research showed that family members of the patients with lung cancer were faced with a series of adverse social-economic changes such as separating from common social and leisure activities, losing family income, reducing working hours, leaving jobs, and consequently having a major change in lifestyle [19].

The other concern about maintaining economic health was not to impose medical expenses on the family. The students said that if they were seriously ill, families would have to bear heavy costs for medical treatment and care. In another study, adolescents and young adults with cancer expressed concerns about the impact of their disease on family financial status and tried to protect the family by preventing family economic problems [17].

Achieving goals and success in life was another motivational factor for appropriate nutritional behaviors in students. In this regard, the desire to form a family and be successful in life was one of the goals mentioned by the participants. They were interested in getting married and creating a successful married life, so they asserted that appropriate nutrition would create a beautiful body and transparent skin, while maintaining the health and playing an effective role in choosing the right spouse. In another study, obese adolescents reported problems in finding a spouse [20]. Fear of infertility and impaired pregnancy were among the other important effective factors on nutritional behaviors. The adolescents expressed concerns about infertility, delayed pregnancy, weak children, and stillbirth due to inappropriate nutritional behaviors. In confirmation of these findings, previous studies reported fear of infertility, fetal harm, and excessive fetus enlargement in pregnancy were the main causes of concern among adolescents and young adults with cancer and diabetes [21, 22].

Another goal proposed by the students was achieving career goals in the future. They tended to find a desired job and believed that in order to achieve some specific occupations (such as pilots and medical staff), they should have an appropriate physical appearance, which can be achieved by appropriate nutritional behaviors. Fear of unemployment due to the disease complications was another factor mentioned by the students. They believed that nutrition-related diseases could cause complications such as disability and loss of body organs and prevented the individuals from achieving their desirable jobs. According to the literature, young people also believed that having a chronic disease could limit their opportunities of choosing and achieving a job. Fear of amputation and future consequences were also among the other concerns mentioned by young adults with diabetes [21, 23].

## 5. Limitations

In this study, the data were collected using individual interviews; no focus-group discussions were conducted. However, focus-group discussions among adolescents can lead them to think actively, participate in the topic, and raise their viewpoints. Therefore, in future studies, application of the focus-group discussion method can provide more comprehensive information to explain the motivational system in the healthy and safe nutrition behavior of Iranian adolescent girls.

## 6. Conclusion

This study provided rich and deep information about the motivational system of Iranian adolescent girls to perform healthy and safe nutrition behavior. Adolescents proposed a variety of factors that could play effective roles in provoking them to adopt nutritional behaviors. Given the nutritional challenge for adolescents' health, identification of the motivational factors can increase the success of interventions designed to promote nutritional behaviors in adolescents.

## Figures and Tables

**Table 1:** Demographic characteristics.

Variables	Mothers	Fathers
	(*N* = 42)	(*N* = 42)
*Age*		
Range	34-43	35-52
Mean ± SD	38.02 ± 2.60	42.57 ± 4.84
*Education: n (%)*		
Illiterate and primary school	3 (7.1)	2 (4.8)
Secondary school	6 (14.3)	5 (11.9)
High school and diploma	21 (50)	17 (40.5)
Academic	12 (28.6)	18 (42.9)
*Occupation: n (%)*		
Housewife	33 (78.6)	—
Self-employed	4 (9.5)	24 (57.1)
Employee	5 (11.9)	15 (35.7)
Worker	—	3 (7.1)
*Number of children*	Girl	Boy
Range	1-3	0-4
Mean ± SD	1.57 ± 0.59	0.52 ± 0.80
*Family income in month (tomans)*		
Range	800000-6500000	
Mean ± SD	3530952.380 ± 1535750.884	

**Table 2:** Categories, subcategories, and sub-subcategories of the motivational factors in the healthy and safe nutrition behavior of Iranian adolescent girls.

Construct	Categories	Subcategories	Sub-subcategories
Motivational factors in the healthy and safe nutrition behavior	(1) Maintaining health and social functions	(1.1) Desire to have an appropriate look and appearance	(1.1.1) Desire to have fitness (1.1.2) Desire to have beautiful skin and hair
		(1.2) Fear of diseases and their complications	(1.2.1) Fear of medical complications following the diseases (1.2.2) Fear of disrupting social functions
	(2) Maintaining the family's mental and economic health	(2.1) Maintaining mental health of family	(2.1.1) No change of mood in the family (2.1.2) No disturbance in family functions
		(2.2) Maintaining the economic health of the family	(2.2.1) Not causing job problems for the family (2.2.2) Not imposing medical expenses on the family
	(3) Achieving goals and success in life	(3.1) Desire to form a family and be successful in life	(3.1.1) The desire to marry and be successful in married life (3.1.2) Fear of infertility and disorder in pregnancy
		(3.2) Achieving the future career goals	(3.2.1) Desire to have a desired job (3.2.2) Fear of unemployment due to disease complications

## Data Availability

All data and materials generated in this study are included in this article, but if necessary, other data will be made available from the responsible author upon request.
